# LGBT+ Health Teaching within the Undergraduate Medical Curriculum

**DOI:** 10.3390/ijerph16132305

**Published:** 2019-06-28

**Authors:** Jessica Salkind, Faye Gishen, Ginger Drage, Jayne Kavanagh, Henry W. W. Potts

**Affiliations:** 1 Department, University College London Medical School, London WC1E 6JL, UK; 2 Royal Free London NHS Foundation Trust, London NW3 2QG, UK; 3 Groundwork London, London SE1 7QZ, UK; 4 UCL Institute of Health Informatics, London NW1 2DA, UK

**Keywords:** LGBT, gay, lesbian, transgender, undergraduate medical education, decolonizing the curriculum, medical education, curriculum development

## Abstract

Introduction: The lesbian, gay, bisexual, and transgender (LGBT+) population experience health and social inequalities, including discrimination within healthcare services. There is a growing international awareness of the importance of providing healthcare professionals and students with dedicated training on LGBT+ health. Methods: We introduced a compulsory teaching programme in a large London-based medical school, including a visit from a transgender patient. Feedback was collected across four years, before (n = 433) and after (n = 541) the session. Student confidence in using appropriate terminology and performing a clinical assessment on LGBT+ people was assessed with five-point Likert scales. Fisher exact tests were used to compare the proportion responding “agree” or “strongly agree”. Results: Of the students, 95% (CI 93–97%) found the teaching useful with 97% (96–99%) finding the visitor’s input helpful. Confidence using appropriate terminology to describe sexual orientation increased from 62% (58–67%) to 93% (91–95%) (Fisher *p* < 0.001) and gender identity from 41% (36–46%) to 91% (88–93%) (*p* < 0.001). Confidence in the clinical assessment of a lesbian, gay or bisexual patient increased from 75% (71–79%) to 93% (90–95%) (*p* < 0.001), and of a transgender patient from 35% (31–40%) to 84% (80–87%) (*p* < 0.001). Discussion: This teaching programme, written and delivered in collaboration with the LGBT+ community, increases students’ confidence in using appropriate language related to sexual orientation and gender identity, and in the clinical assessment of LGBT+ patients.

## 1. Introduction

In many parts of the world, the political and social progress of recent decades has significantly improved the lives of people who are lesbian, gay, bisexual, and transgender (LGBT+, with the “+” indicating inclusion of all sexual and gender minority identities). Despite this progress, even in countries with the most robust legal equality for the LGBT+ population, there remain significant health and social inequalities. Multiple international studies have consistently found higher rates of depression, anxiety, alcohol and drug use, self-harm and suicide, alongside worse physical health outcomes in the LGBT+ community [1,2,3,4]. These have been linked to social inequalities stemming from homophobia, biphobia, and transphobia [5]. There is evidence that these inequalities extend to those being treated and working within healthcare systems. For example, in a survey of over 5000 staff within the UK National Health Service (NHS), 25% of staff had heard homophobic language at work and 20% had heard transphobic language at work [6]. Transgender patients have reported being addressed by the wrong names and pronouns, and feeling that they have to educate healthcare professionals [7]. 

In order to address these inequalities, international organisations including the World Health Organisation [8] and the Association of American Medical Colleges [9] have called for dedicated teaching on LGBT+ health for healthcare students and professionals. Consequently, some healthcare programmes have introduced teaching on LGBT+ health. An example of a comprehensive teaching programme is that offered by the University of Louisville School of Medicine, who have introduced a 50.5 hour integrated programme including a patient panel, with encouraging initial outcomes in terms of reduced implicit bias based on sexuality [10]. A recent systematic review of 15 LGBT+ teaching programmes (seven of which were medical schools) found improvements in knowledge, attitudes and/or practice towards LGBT+ people, however they did not evaluate whether these translated into improvements in the care of LGBT+ patients. The authors reported that the content of the teaching varied between programmes, but in general there was less focus on the specific issues faced by those who are transgender/non-binary and programmes often had no or minimal involvement of LGBT+ people themselves [11]. 

With this in mind, we introduced a half-day programme for all fifth year medical students (in their penultimate year of the undergraduate course) in a large London medical school. The year before, a pilot programme had been introduced that covered sexual orientation only and was led by senior medical students with no input from LGBT+ patient visitors. The positive feedback to this initial session led to the expansion of the programme. The expanded programme was strongly based on the input of LGBT+ people with an equal focus on sexual orientation and gender identity. The teaching programme aimed to enable students to understand and explore the impact of prejudice and discrimination on LGBT+ people and to consider how medical students and doctors can promote their health and wellbeing.

## 2. Methods

### 2.1. Setting and Context

This half-day teaching programme was embedded within a compulsory fifth year summative teaching week, bringing together key themes from the year’s teaching, including obstetrics and gynaecology, paediatrics, general practice, care of the older patient, psychiatry, and palliative care. International guidance recommends embedding LGBT+ teaching throughout the curriculum [12], and this fifth year teaching complements a lecture for first year students on gender identity and sexual orientation, and further teaching on transgender medicine within the ‘Child Health’ module. 

### 2.2. Development of Materials

The teaching materials were developed over several months by Jessica Salkind a junior doctor, using an iterative technique, with input and feedback from self-identifying LGBT+ people. They have subsequently been updated each year in response to student and teacher feedback. As discussed above, many teaching programmes of this kind have placed more onus on sexual orientation, therefore significant effort was made to gain input from transgender and non-binary people who generously shared their stories and helped construct the clinical scenarios to make them as realistic as possible. 

### 2.3. Teaching Session Structure

The programme was structured as follows: (1) A 45 minute lecture incorporating key background knowledge, terminology, LGBT+ inequality, legal protection for LGBT+ people and professional guidance; (2) a 45 minute session with a patient visitor who identifies as transgender with the opportunity for students to ask questions about their experiences of accessing healthcare services as well as more general questions; (3) a 1.5 hour seminar to work through four clinical scenarios and generate best practice advice for making services LGBT+ inclusive. 

### 2.4. Facilitators

While other models have used senior medical students to facilitate this type of teaching [13,14], within this programme, self-identifying LGBT+ junior doctor facilitators were selected for a number of reasons. Junior doctors have more clinical experience, allowing them to integrate clinical learning into the sessions and answer questions confidently, while still being relatable to students. In addition, there are fewer issues around confidentiality if they choose to share stories about their own experiences, and it has proven easier to ensure the sustainability of the programme. As the majority of the facilitators are cisgender (and identify as lesbian, gay or bisexual), they received additional training on transgender/non-binary issues which may be outside their personal experience. All facilitators had the opportunity to spend time with and learn from the patient visitors. They received a literature pack prior to the teaching with guidance on group facilitation, including what to do if problems occurred, such as disagreement between students or how to handle potentially offensive and/or upsetting comments. They also received guidance from senior university staff with experience of hosting patient visitors in medical student teaching. 

### 2.5. Patient Visitors 

The patient visitors, who all identify as transgender/non-binary, were recruited through personal networks, LGBT+ national conferences and via social media. The visitors were provided with written guidance, asking them to share their stories of using healthcare services, to explain to students both positive and negative aspects of care they have had and identify times when things were done particularly well or could have been done better. Prior to the teaching, teaching staff discussed the possible impact of sharing potentially distressing personal stories with an unknown group with each visitor. Each group facilitator met their visitor on the day, prior to the teaching, and senior staff were on hand to offer support to visitors if they wished to debrief afterwards, as well as signposting to external sources of support if needed. Three visitors were invited per session to enable smaller discussion groups (maximum 30 students per group). Students were encouraged to think about potential questions for the visitor in advance. Each visitor was asked about their preferred name, pronouns and whether there were any topics that they did not want to be asked about before the session.

### 2.6. Ethical Approval

The UCL Research Ethics Committee approved the anonymised pre and post-session questionnaires. Project ID: 4415/002.

### 2.7. Funding 

The programme was awarded a £1470 “Liberating The Curriculum” grant by the University, designed to increase teaching related to equality, diversity and inclusion themes. This money was used to pay for facilitator travel costs, and to pay the visiting speakers for their time and travel costs. Following the positive feedback for programme, these costs are now met by the Medical School. 

### 2.8. Questionnaire Design

An anonymous paper-based questionnaire was given to students before and after the session, using a series of statements with a five-point Likert scale from “strongly disagree” to “strongly agree”. This assessed their views on the importance of the teaching, their confidence in using appropriate language related to sexual orientation and gender identity, and their confidence in taking a history and examining a lesbian, gay or bisexual patient, and a transgender patient. Other models have used similar scales to evaluate self-perceived confidence in clinical assessment of LGBT+ patients [14]. In addition, the post-session questionnaire, completed directly after the session, explored whether the session was useful and whether the visitor had enhanced students’ understanding, with a free-text option for further comments. 

Further face-to-face feedback was gathered informally after each session with all visitors and facilitators. Utilising Quality Improvement methodology, a plan-do-study-act cycle approach was taken, using feedback to make rapid changes to content and structure between consecutive sessions and/or days, and asking visitors and facilitators to evaluate those changes, for example, a role play scenario was introduced in response to a number of free text comments.

## 3. Results

Across 2016–2019, 92, 81, 125, and 135 people respectively (433 total) completed the pre-session questionnaire, and 119, 84, 162, and 176 people respectively (541 total) completed the post-session questionnaire (Table 1). To ensure anonymity, responses were not linked to individuals and therefore paired analyses are not possible. Data were combined across the four years, using Fisher exact tests to compare the proportion responding ‘agree’ or ‘strongly agree’ before and after the session. 

Prior to the session, a small proportion of the group, 9% (CI 6–12%) did not agree with the idea that LGBT+ people face health and social inequalities which are relevant to clinical practice. After the session, this proportion decreased to 1% (1–3%) (*p* < 0.001). There were significant improvements in confidence using appropriate terminology to describe sexual orientation from 62% (58–67%) pre-session to 93% (91–95%) post-session (*p* < 0.001). There was a larger improvement for confidence in using appropriate terminology to describe gender identity, where there was a lower starting confidence pre-session: from 41% (36–46%) to 91% (88–93%) post-session (*p* < 0.001).

Pre-session, 75% (71–79%) of students were confident in the clinical assessment of a lesbian, gay or bisexual patient (including using appropriate language), which increased to 93% (90–95%) post-session (*p* < 0.001). As with terminology, a bigger change was seen with regards to the clinical assessment of a transgender patient, where there was a low initial confidence of 35% (31–40%) pre-session, increasing to 84% (80–87%) post-session (*p* < 0.01).

Overall, nearly all students (95%; CI 93–97%) found the teaching session useful and felt that the visitor had enhanced their understanding of the topics covered in the session (97%; CI 96–99%). In the most recent year, only one student out of 176 did not report the session as useful.

The free text comments were generally positive, with the session described as “a really informative session (which) highlighted the complexity of these issues which I hadn’t previously considered” and “something that isn’t taught anywhere else in our curriculum, but highly relevant & important to be educated on”. Many comments referred directly to the visitors, “I found having a chance to speak with the transgender visitor extremely helpful & insightful”, but described wanting more time to ask questions: “could spend even longer discussing issues with them”. The feedback from the patient visitors was similarly positive, with one person describing it as “a very empowering experience and more importantly, one that will hopefully help shaping their future attitude towards transgender people, when it comes to it”.

## 4. Discussion

The results showed marked improvements across all five questions and very positive assessments of the session’s usefulness and the value of having the visitor.

There was a significant improvement in confidence in using appropriate terminology to describe people who are LGBT+. This is important, as uncertainty regarding appropriate terminology may underlie the reports of inappropriate and potentially offensive language being used by healthcare professionals to describe those who are LGBT+ [6]. Within the clinical scenarios, students were encouraged to describe ways in which they could challenge inappropriate or offensive language if they overheard it during their clinical placements, for example via the medical school’s raising concerns system. The reported increase in confidence in taking a history and examining patients who are LGBT+ is key to ensuring equitable access to healthcare regardless of sexual orientation and gender identity, in line with the Equality Act [15] in the UK. Transgender people have reported being asked inappropriate questions, for example, about their plans for genital surgery when presenting with an unrelated medical problem, and of their gender identity overshadowing an underlying, unrelated problem [7]; this may be mitigated against by a full and appropriate clinical assessment. 

The biggest improvements subsequent to the teaching related to describing gender identity and interacting with transgender people, due to initial lower confidence compared with describing sexual orientation and interacting with lesbian, gay and bisexual people. These findings reflect published data that students are more comfortable discussing issues related to sexual orientation than gender identity [16]. The authors propose that the increase in confidence around gender identity may be, in part, due to the time spent with the transgender visitor, with nearly all (97%; CI 95–98%) reporting that the visitor enhanced their understanding of the topics covered, a feeling echoed in the free text comments. Confidence in taking a history and examining a transgender patient, although greatly improved, was the only question that received less than 9 out of 10 positive responses after the session, with 84% agreeing they would feel confident, suggesting this area remains challenging for some students.

To the best of our knowledge, this programme is unique in offering all students within a medical school year cohort the opportunity to hear the stories and ask questions of a visitor who is transgender. The benefit of inviting visitors seems to be two-fold. Intergroup contact theory predicts that exposure to LGBT+ people can reduce prejudice, and there is growing evidence for this in similar settings to this one [17,18,19]. For all students, it is likely that having the opportunity to ask questions about a group they have potentially had little contact with could reduce discomfort. Evidence from Louisville showed that after an event involving interaction between healthcare professionals and transgender community members, the healthcare professionals felt more confident to work with transgender patients [20]. Furthermore, the real-life expertise provided by the visitors, is likely to provide the most valuable and valid best practice advice for students. This best practice advice is also incorporated into the four clinical scenarios, created from the amalgamation of real life stories shared by LGBT+ patients, as it has been suggested that hypothetical cases can lack the complexity of real clinical cases [21]. In this way, the whole teaching programme directly reflects the lived experiences of LGBT+ people who have been treated recently within the UK NHS. By delivering this teaching in the fifth year, the students have already had sufficient clinical experience and generic history-taking and examination skills to engage meaningfully with the clinical scenarios, consider best practice and contribute their own stories from their clinical placements. By incorporating this training within the core curriculum, its sustainability has been ensured. The model could be easily transferred to other healthcare training settings. While in a large teaching hospital in London, there is a baseline of acceptance towards LGBT+ people and robust legal equality, training of this kind could have even more impact in settings where this is not the case.

A limitation of this work is that, as with other teaching delivered within the same summative teaching week, the teaching session had a relatively low attendance rate of about one third of the year cohort despite it being a mandatory session. It is not currently possible to assess whether this represents selection bias, for example, with those students who are LGBT+ themselves, or those who have friends or family who are, being more likely to attend [22] or if this simply represents a diligent cohort of students who attend all teaching sessions. Efforts are being made by the university to increase attendance through a sign-in sheet. As acknowledged in other work of this kind [11], it is not possible to determine if students’ immediate feedback will translate into longer-term change in attitude towards LGBT+ people or a change in clinical practice and improvement in clinical care. The next step is to incorporate LGBT+ scenarios into medical student examinations. There is the potential to use a validated tool to assess students after the teaching—evidence has recently been provided for the lesbian, gay, bisexual, and transgender development of clinical skills scale (LGBT-DOCSS) [23].

## 5. Conclusions

This programme has been positively evaluated by medical students and greatly increases their confidence in using appropriate language related to sexual orientation and gender identity, and in performing clinical assessments on patients who are LGBT+. Further research is required to measure whether improved student confidence translates into improved patient care for the LGBT+ community. This is key for a group with proven healthcare disparities who may disengage from healthcare services if not treated with understanding and respect. 

## Figures and Tables

**Table 1:** Pre and post-session questionnaire results (2016–2019 data pooled).

Question	Pre		Post		Fisher Exact Test *p*
	% Agree/Strongly Agree	95% CI	% Agree/Strongly Agree	95% CI	
LGBT+ people face health and social inequalities which are relevant to clinical practice	395/433 (91%)	88%–94%	533/540 (99%)	97%–99%	<0.001
I feel confident using appropriate terminology to describe sexual orientation.	270/433 (62%)	58%–67%	504/540 (93%)	91%–95%	<0.001
I feel confident using appropriate terminology to describe gender identity.	176/432 (41%)	36%–46%	490/541 (91%)	88%–93%	<0.001
I would feel confident taking a history from and examining a lesbian, gay or bisexual patient, including using appropriate language.	326/433 (75%)	71%–79%	501/541 (93%)	90%–95%	<0.001
I would feel confident taking a history from and examining a transgender patient, including using appropriate language.	153/433 (35%)	31%–40%	453/541 (84%)	80%–87%	<0.001
I found the session useful.			516/541 (95%)	93%–97%	
The visitor enhanced my understanding of topics covered in the session.			527/541 (97%)	96%–99%	

grey lines: These questions evaluating the teaching were asked in the post-session questionnaire only, therefore a Fisher test could not be applied.

## Glossary Terms

Sexual orientation	describes who a person is sexually attracted to.
Homosexual/gay/lesbian	a person who is sexually attracted to people of the same gender.
Bisexual	a person who is sexually attracted to people of the same gender and another gender/other genders.
Gender identity	how a person identifies in terms of being a man, a woman, both, neither or another identity altogether.
Cisgender /cis	a person whose gender identity is consistently congruent with the sex they were assigned at birth.
Transgender/trans	a person whose gender identity is not consistently congruent with the sex they were assigned at birth.
Non-binary	any gender identity outside of exclusively ‘man’ or ‘woman’; a non-binary person may or may not identify as transgender.
Homophobia/biphobia/transphobia	hatred and/or intolerance of people who are homosexual/bisexual/transgender.
